# Comment on Naef, R.; Acree, W.E., Jr. Calculation of the Three Partition Coefficients logPow, logKoa and logKaw of Organic Molecules at Standard Conditions at Once by Means of a Generally Applicable Group Additivity Method. *Preprints* **2023**, 2023120275

**DOI:** 10.3390/molecules29040892

**Published:** 2024-02-18

**Authors:** Robert J. Meier

**Affiliations:** Independent Researcher, 52525 Heinsberg, Germany; r.meier@planet.nl

Next to the paper referred to in the title [1], Naef and Acree have published a series of papers involving the group contribution (GC) method, which has been applied to a larger variety of physico-chemical properties all published in *Molecules* [2,3,4,5,6,7,8]. The GC method is based on the assumption that some aspects of chemical groups are the same in many different molecules. The GC method is a so-called data-driven model, with experimental data being used to parametrize the model. A molecular property of interest is the sum (additive) of the individual properties of molecular fragments j, with Nj being the number of times the fragment j occurs in the molecule, i.e.,


(1) $${\Delta H}_{f}=\sum_{j=1,N}{Nj\cdot \Delta H}_{f}(j)$$

We talk more specifically about the group additivity method, as in the title of the paper by Naef and Acree.

Regarding the former paper by Naef and Acree on the heat of formation of organic molecules [2], we criticized this publication as the authors had grossly overlooked very many relevant publications over the last 25 years [9]. There have been numerous papers by several excellent groups that reported significant progress on this topic with partly excellent results. Interestingly, Naef and Acree did not provide a rebuttal to the Comment [9]. We were more astonished when seeing the latest contribution by Naef and Acree [1], which addresses the octanol–water partition coefficient log Kow. The prediction of log Kow, or alternatively log P, has been of considerable interest in various fields and was therefore often studied; a huge number of reports as well as software tools are available. Similarly to the case of the heat of formation, Naef and Acree state now that their results ‘clearly outperform Klopman’s results’; however, Klopman’s paper dates back to 1994! Apart from many individual papers, also-important review papers [10,11] are simply not quoted, and do not discuss comparisons of their results to the state of the art, which should be key in any scientific publication. Furthermore, there is ample literature not quoted by Naef and Acree, and there is a larger variety of software tools both freely available as well as commercial ones (a few are mentioned by Naef and Acee, but no proper analysis is provided). In 2002, Marrero and Gani reported a GC-based study on log Kow [12] with the result, ‘The group-contribution values were calculated by linear regression analysis using a data set of 9560 values for *K*_ow_. The data set included compounds ranging from C3 to C70, including large and heterocyclic compounds. Compared to other currently used group-contribution methods, the new methods make significant improvements in accuracy with logarithm-unit average absolute errors of 0.24 for *K*_ow_’. In comparison, Naef and Acree reported results based on 3332 molecules (about 1/3 of the Marrero–Gani work) with a standard deviation of 0.42. Firstly, it needs to be mentioned that the earlier value from Naef and Acree was 0.51, whereas the lower value of 0.42 is the result of the removal of 122 molecules (3.5% of the total set) from the parameter computation for which the experimental value deviates by more than three times the value of S. As the S(tandard) D(eviation) is more sensitive to outliers, the results of Marrero–Gani and Naef and Acree might still be close; however, the latter is not better, whereas the former result dates back to 2002. The Marrero–Gani work was based on three times as many species, which implies a larger range of validation and an absolute average error, which compares well with the experimental error that is typically in the range of 0.2–0.4 log units [13]. At a later stage, the results from the same group reported a standard deviation of 0.61 and an averaged absolute deviation of 0.45 log units based on 12,193 different molecules. The results and many more properties were reported in the thesis of Amol Shivajirao Hukkerikar (2013), which is freely available [14], as well as in a paper [15]. We wish to emphasize that only looking at the standard deviation or a similar quantity is insufficient; the number of different molecules is crucial regarding the proven applicability range.

A more recent paper considers log Kow with deep learning techniques [13]. In addition, interestingly, the software tool developed has been made available via the Supplementary Material. Different DNN models were explored based on a data set comprising 14,050 chemicals. The authors have considered both neutral as well as ionic species. The data set itself was said to be heterogeneous, including many different classes of chemical compounds. After the exclusion of some identified erroneous data points, a root mean square error (rmse) of 0.47 and a very small standard deviation was reported; for details, refer to Ref. [13].

There are more works on log Kow that are of relevance. In summary, it seems justified to state that whereas Naef and Acree claim that their method ‘has proven its unmatched versatility in the equally reliable prediction of up to now 19 physical, thermodynamic, solubility-, optics-, charge-, and environment-related molecular descriptors based on a common group-additivity method’, this is in such a general way that has not been supported by the literature of the last 20 years. The statement ‘enabled their prediction for nearly 29,500 molecules’ merely means that the tool developed based on much fewer species is simply applied to 29,500 molecules, whereas other existing methods have performed, as proven, with respect to that many species.
